# Propensity-matched analysis of the impact of saphenous vein graft external stenting on clinical outcomes in coronary bypass surgery: The RESTART study

**DOI:** 10.1016/j.xjon.2025.09.048

**Published:** 2025-10-24

**Authors:** Luca P. Weltert, Eric A. Secemsky, Gil Bolotin, Tom Friedman, Paolo Centofanti, Viviana Sebastiano, Samuel Fusca, Sigrid E. Sandner, Marija Pljakova, Stefanos Demertzis, Tiziano Torre, John T. Donovan, Ivar Friedrich, Siling Li, Marcus Flather, Stephen Gerry, David P. Taggart

**Affiliations:** a Department of Cardiac Surgery, European Hospital, Rome, Italy; b Department of Medicine, Harvard Medical School, Boston, Mass; c Smith Center for Outcomes Research, Beth Israel Deaconess Medical Center (BIDMC), Boston, Mass; d Department of Cardiac Surgery, Rambam Health Care Campus, Haifa, Israel; e Cardiac Surgery Division, Mauriziano Hospital, Turin, Italy; f Department of Cardiac Surgery, Medical University Vienna, Vienna, Austria; g Cardiac Surgery, Cardiocentro Ticino, Lugano, Switzerland; h Heart & Thoracic Surgery, Herzzentrum Trier, Krankenhaus der Barmherzigen Bruder, Trier, Germany; i Cardiac Surgery, CardioClinic Köln, Köln, Germany; j Cardiovascular & Metabolic Health, University of East Anglia, and Norwich University Hospital, Norwirch, United Kingdom; k Centre for Statistics in Medicine, Nuffield Department of Orthopaedics, Rheumatology & Musculoskeletal Sciences, University of Oxford, Oxford, United Kingdom; l Nuffield Department of Surgical Sciences, University of Oxford, Oxford, United Kingdom

**Keywords:** cardiac surgery, coronary artery bypass grafting (CABG), vein graft external support device, coronary artery disease, coronary heart disease in adults, clinical outcomes, MACCE

## Abstract

**Objective:**

External saphenous vein graft stenting has been shown to reduce intimal hyperplasia, lumen irregularities, and flow disturbances after coronary artery bypass grafting (CABG). The objective of this study is to evaluate the effect of saphenous vein graft external stenting on clinical outcomes up to 5 years.

**Methods:**

Outcomes for patients who received external vein graft stenting in an international, real-world cohort were compared in a propensity matched analysis with patients from the Arterial Revascularization Trial (ISRCTN46552265). All eligible patients required an internal mammary artery graft to the left anterior descending coronary artery, received at least one vein graft, and survived to discharge. The primary end point was major adverse cardiovascular and cerebrovascular events at 1 year after surgery, consisting of all-cause mortality, myocardial infarction, repeat revascularization, and cerebrovascular accident. Secondary end points included 5-year major adverse cardiovascular and cerebrovascular events with and without stroke and annualized target vessel revascularization.

**Results:**

In total, 789 treated and 2205 control patients were included. At 1 year after CABG, the weighted hazard ratio comparing outcomes between treated and control patients was 0.60 (90% confidence interval, 0.38-0.94, *P* = .03). The benefits associated with external stenting for the composite outcome persisted through 5 years’ post-CABG (hazard ratio, 0.70; 95% confidence interval, 0.51-0.98, *P* = .04). Annual target vessel revascularization rates in vein grafts were significantly lower in the venous external support cohort at 2 to 5 years after surgery (*P* = .009-.03).

**Conclusions:**

The current study demonstrates that external vein graft stenting is associated with a significantly lower risk of experiencing adverse clinical outcomes up to 5 years after surgery compared with standard of care.

## Graphical Abstract

**Figure:**
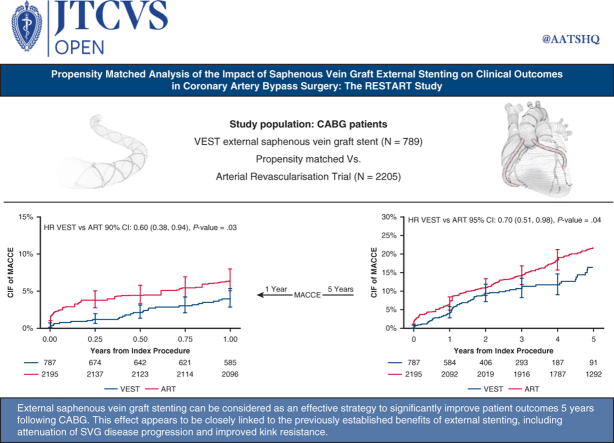

**Figure:**
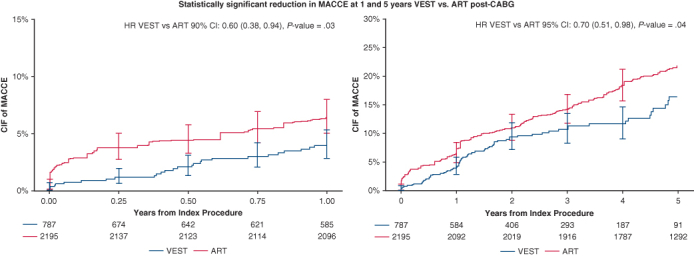
Statistically significant reduction in MACCE at 1 and 5 years with VEST versus ART post-CABG.

**Central Message**

External stenting of SVGs with VEST during CABG significantly reduces MACCE at 1 and 5 years, mainly by reducing vein graft−related revascularizations.

**Perspective**

SVG failure remains a major limitation to the long-term success of CABG. This large, propensity-matched analysis of real-world patients shows that external stenting with VEST improves long-term outcomes, with significant reductions in MACCE and TVR through 5 years. These findings support VEST as an effective strategy to enhance graft durability and patient clinical outcomes after CABG.

Coronary artery bypass grafting (CABG) remains the gold standard treatment for revascularization of multivessel coronary artery disease.^1^ CABG typically involves arterial grafting of the left anterior descending artery with an internal mammary artery (IMA), as this conduit has demonstrated superior longitudinal patency and outcomes.^2^ In addition, other arterial and saphenous vein grafts (SVGs) are commonly used as conduits for additional diseased coronary vessels. SVGs, however, are subject to early, midterm, and late failure, with only 50% of SVGs patent at 10 years.^3^^,^^4^

Mechanisms of failure of SVGs have been previously explored and defined. Periprocedural and early (ie, <30 days) SVG failure, which ranges between 8% and 12%, is primarily attributed to SVG kinking, technical errors, vessel trauma, or poor coronary vascular bed.^5^ At the same time, the pathologic process of adverse SVG remodeling begins immediately after implant, with intimal hyperplasia triggered by exposure to the hemodynamics of the arterial system.^6^^,^^7^ With the exception of statins and beta-blockers, pharmacotherapy has not been successful at reducing this burden of intimal hyperplasia or significantly improving conduit patency.^8^ Conversely, translational studies have demonstrated that external scaffolding of the SVG can alter the hemodynamic vascular injury and retard the cadence of progressive SVG disease.^9^^,^^10^ This approach has been demonstrated to be safe and feasible and to successfully reduce SVG disease in randomized control trials^11^, ^12^, ^13^ as well as routine clinical practice.^14^ Real-world clinical outcomes of external SVG stenting compared with matched controls are needed to extend these comparative outcome results. In this study, we aimed to evaluate the impact of an external stent for SVG compared with routine CABG on major adverse cardiovascular and cerebrovascular events using a propensity matched design.

## Methods

### Patients and Design

**Figure 1:**
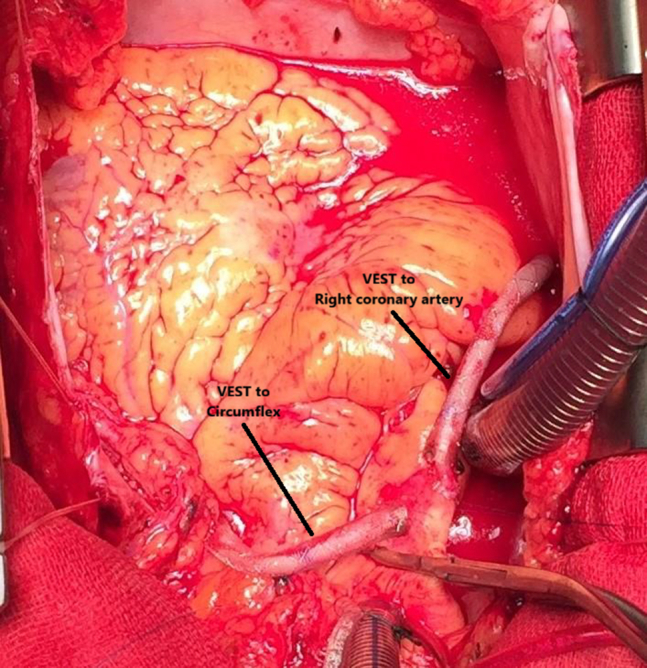
Two saphenous vein grafts with VEST external stents before chest closure. *VEST*, Venous external support.

This multicenter, observational cohort study assessed the effectiveness of an external SVG stent (venous external support, or VEST; Vascular Graft Solutions; Figure 1) in patients undergoing CABG. Since its commercial introduction in 2015, VEST has been implanted in more than 9000 CABG patients. Data were systematically collected from 6 international centers for patients treated with VEST between 2015 and 2024, using standardized case report forms aligned with the Arterial Revascularization Trial (ART) trial database. In addition to the systematic review of medical records, all patients were proactively followed for latest clinical outcomes information. Collected variables included patient demographics, comorbidities, postoperative pharmacologic management, and detailed clinical outcomes, with a particular focus on repeat revascularization events up to 5 years postoperatively. Consecutive patients who received 1 or more external SVG stents were included.

For comparative analysis, a historical control group was derived from ART (ISRCTN46552265).^2^ ART was a randomized controlled trial that enrolled 3102 CABG patients in 7 countries, assigning them to either single or bilateral IMA grafting, with clinical follow-up extending to 10 years. ART was approved by the relevant ethics committees, and patients provided written informed consent. ART data were previously published multiple times^2^^,^^15^^,^^16^ and were applied in the current study under a data-sharing agreement according to the online appendix nejmoa1808783_data-sharing.^2^ Patients who received VEST provided informed consent, as applicable, in accordance with local ethics committee requirements. In centers requiring written consent, patients were informed that study findings could be published, provided no identifiable personal information was disclosed. RESTART study was approved by the Committee on Clinical Investigations at Beth Israel Deaconess Medical Center (BIDMC; approval number 2024P000272, dated June 3, 2024).

Patients were eligible if they received an IMA graft to the left anterior descending coronary artery, at least 1 vein graft, and survived to hospital discharge. Patients undergoing concomitant valve surgery or before CABG were excluded from analysis. In addition, patients who were treated in an Organisation for Economic Co-operation and Development country were considered eligible.

### End Points and Definitions

The primary end point was the cumulative incidence of major adverse cardiovascular and cerebrovascular events (MACCE) at 1 year after CABG surgery, defined as a composite of all-cause mortality, any myocardial infarction (MI) >3 days postprocedure, any revascularization—including percutaneous coronary intervention (PCI) or repeat CABG—and cerebrovascular accident (CVA). Secondary end points included MACCE, the cumulative incidence of individual components of MACCE, target vessel revascularization (TVR), and annualized TVR rate through 5 years. Given that external stenting is not expected to influence the incidence of CVA, a secondary composite end point of major adverse cardiac events (MACE) was defined, comprising all-cause mortality, MI, and any repeat revascularization at 5 years after CABG.

Outcome definitions for the patients in the VEST group followed those of the ART study. Thus, MI was diagnosed on the basis of the presence of at least 2 of the following criteria: (1) unequivocal electrocardiogram changes; (2) elevation of cardiac enzyme(s) above twice the upper limit of normal or diagnostic troponin rises; and (3) chest pain typical for acute MI that lasted more than 20 minutes (as defined in the ART trial). Periprocedural MIs occurring within 3 days of CABG were excluded, following the ART definition of periprocedural MIs extending out to 72 hours post CABG. CVA was defined as a new focal neurologic deficit thought to be vascular in origin with signs and symptoms lasting more than 24 hours. TVR was defined as any revascularization to a previously vein grafted coronary region, either involving the vein graft itself or the native coronary vasculature subtending the cardiac territory. Events were identified through scheduled follow-up and medical records and then adjudicated according to the aforementioned predefined definitions.

### Surgical Method for External SVG Stenting

The external stent is a tubular scaffold composed of braided, deformable cobalt-chromium wires. Available in multiple dimensional configurations, it features a flexible architecture designed to accommodate placement over the autologous SVG. This flexibility allows for precise adjustment of both length and diameter to ensure optimal graft coverage while avoiding interference with the anastomotic sites. After vein graft harvesting and preparation, the stent is deployed intraoperatively and positioned over the vein graft before anastomotic completion. No additional fixation is required, as the stent remains secured between the anastomoses.

### Statistical Methods

**Figure E1:**
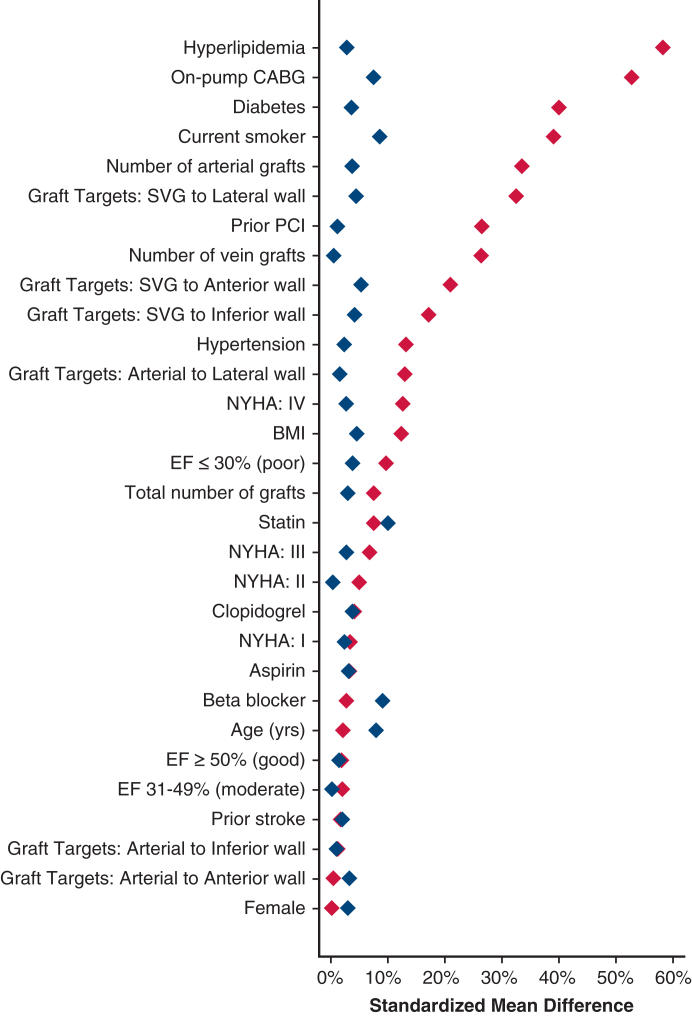
Love plot depicting a list of covariates and the standardized mean differences before (*red*) and after (*blue*) weighting. *CABG*, Coronary artery bypass grafting; *SVG*, saphenous vein graft; *PCI*, percutaneous coronary intervention; *NYHA*, New York Heart Association; *BMI*, body mass index; *EF*, ejection fraction.

The inverse probability treatment weighting to estimate the average treatment effect on the treated method was used as the primary analytic tool to correct for potential confounding due to observed characteristics. The inverse probability treatment weighting to estimate the average treatment effect on the treated approach was conducted in 2 stages. First, eligible patients were identified from the databases on the basis of predefined criteria. Logistic regression was used to estimate the propensity score (p), incorporating multiple demographic, baseline, surgical, and postoperative variables. A love plot detailing the complete list of variables is provided in Figure E1. Variables were selected on the basis of clinical relevance for outcomes, established literature on related risk factors, availability in both datasets, and baseline imbalances (standardized mean difference [SMD] >10%) requiring adjustment. Missing values were imputed using median values for continuous variables and mode values for the categorical variables. The weighting variable, $w_{A}$, was defined as 1 for individuals in the treatment group and p/(1−p) for those in the control group. Covariate balance between the treatment and control groups was assessed after the application of weights, with an SMD of >10% suggesting imbalance. Subsequently, the weighted dataset was linked to outcomes data to assess both primary and secondary end points. Absolute risk difference was calculated with 2-sided 90% confidence interval (CI), and *P* values were derived using the bootstrap method.^17^ Cox regression models, with group membership as the sole covariate, were used to estimate the hazard ratio of MACCE through 1 year, comparing the external stent group to the routine CABG group. In these regression models, each subject's weight was incorporated to adjust for any residual imbalance in patient characteristics.

Similar methods were used for the secondary end points. For all-cause mortality, the Kaplan-Meier method was used to estimate the cumulative incidences, and Cox regression was performed to estimate the hazard ratio (HR). For nonfatal outcomes including any MI, any revascularization, TVR and CVA, the competing risk of all-cause mortality was accounted for using the Cumulative Incidence Function and Cox regression with the Fine-Gray method.^18^ The subdistribution HR was reported.

In addition, TVR was examined on an annual basis between the treatment and control cohorts. Poisson regressions were used with the number of events per-patient included as the response variable, the natural log of length of follow-up time included as the offset. All analyses were performed with SAS, version 9.4 (SAS Institute).

## Results

### Patient Selection and Weighting

**Figure E2:**
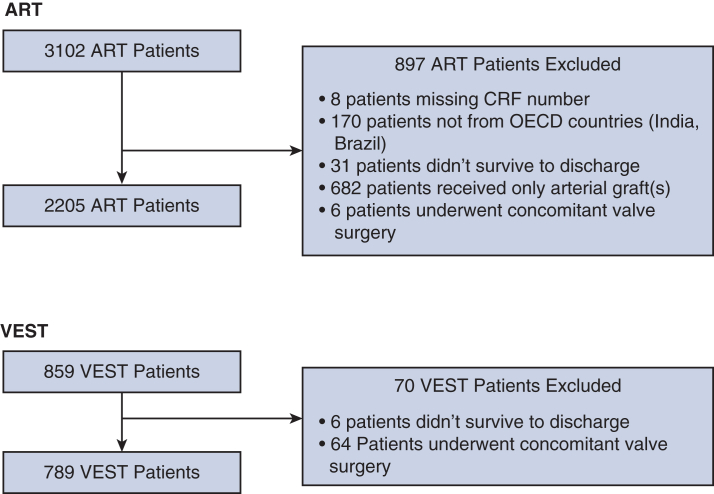
Study flowchart for the creation of the primary analytic cohorts. *ART*, Arterial Revascularization Trial; *CRF*, case report form; *OECD*, Organisation for Economic Co-operation and Development; *VEST*, venous external support.

A total of 3102 patients in the ART cohort and 859 patients in the VEST (external SVG stent) cohort were identified for inclusion in the study. After applying exclusion criteria, the final study populations consisted of 2205 patients in the ART cohort and 789 patients in the VEST cohort. The study flowchart for the cohort is provided in Figure E2. The primary reasons for exclusion in the ART cohort were residence in a non-Organisation for Economic Co-operation and Development country (n = 170; 5.48%) and undergoing complete arterial bypass grafting without use of SVG (n = 682; 21.99%). In the VEST cohort, the most common exclusion criterion was concomitant valve surgery (n = 64; 7.45%).

Before weighting, patients in the VEST cohort exhibited greater rates of diabetes (40.6% vs 22.24%; SMD 40%), tobacco use (30.0% vs 14.1%; SMD 39%), and previous PCI (26.5% vs 15.8%; SMD 26.5%) compared with the ART cohort. Conversely, patients in the VEST cohort had a lower prevalence of hyperlipidemia (74.3% vs 94.5%; SMD −58.1%). Procedurally, VEST-treated patients were more likely to undergo on-pump CABG (86.1% vs 64.0%; SMD 52.7%) and received a greater number of arterial grafts (1.8 ± 0.9 vs 1.5 ± 0.7; SMD 33.8%) compared with patients in the ART cohort (Table 1). After weighting, all patient and procedural characteristics were well balanced between the ART and VEST cohorts, mean age 64 years and 13% female, with no residual imbalances (all SMDs <10%, Table 1).

**Table 1:** Demographic, baseline, surgical, and medical therapy patient characteristics: Weighted and unweighted

Subject characteristics	Unweighted			Weighted		
	VEST (n = 789 subjects)	ART (n = 2205 subjects)	Standardized difference (%)	VEST (n = 789 subjects)	ART (n = 2205 subjects)	Standardized difference (%)
Age, y, mean ± SD	63.5 ± 9.1	63.7 ± 8.8	−2.1	63.5 ± 9.1	64.3 ± 8.8	−8.1
Female, n (%)	102 (12.9%)	286 (13.0%)	−0.1	12.9%	13.9%	−2.9
BMI, mean ± SD	27.8 ± 4.3	28.3 ± 4.0	−12.2	27.8 ± 4.3	27.6 ± 4.0	4.6
Current smoker (within past 3 mo), n (%)	237 (30.0%)	312 (14.1%)	39.0	30.0%	26.2%	8.7
Hypertension, n (%)	658 (83.4%)	1725 (78.2%)	13.1	83.4%	84.2%	−2.1
Hyperlipidemia, n (%)	586 (74.3%)	2084 (94.5%)	−58.1	74.3%	73.1%	2.8
Diabetes, n (%)	320 (40.6%)	493 (22.4%)	40.0	40.6%	42.3%	−3.6
Previous stroke, n (%)	26 (3.3%)	67 (3.0%)	1.5	3.3%	2.9%	2.0
Previous percutaneous coronary intervention, n (%)	209 (26.5%)	348 (15.8%)	26.5	26.5%	26.0%	1.2
Ejection fraction, %, n (%)						
≤30% (poor)	30 (3.8%)	48 (2.2%)	9.6	3.8%	3.1%	3.7
31%-49% (moderate)	164 (20.8%)	475 (21.5%)	−1.9	20.8%	20.9%	−0.2
≥50% (good)	595 (75.4%)	1682 (76.3%)	−2.0	75.4%	76.0%	−1.4
New York Heart Association classification, I-IV, n (%)						
I	277 (35.1%)	739 (33.5%)	3.4	35.1%	36.2%	−2.3
II	355 (45.0%)	1046 (47.4%)	−4.9	45.0%	45.2%	−0.4
III	149 (18.9%)	360 (16.3%)	6.7	18.9%	17.8%	2.7
IV	8 (1.0%)	60 (2.7%)	−12.6	1.0%	0.8%	2.7
On-pump CABG, n (%)	679 (86.1%)	1411 (64.0%)	52.7	86.1%	83.4%	7.4
Total number of grafts, mean ± SD	3.4 ± 0.9	3.3 ± 0.8	7.2	3.4 ± 0.9	3.4 ± 0.8	2.8
Number of vein grafts	1.6 ± 0.7	1.8 ± 0.8	−26.4	1.6 ± 0.7	1.6 ± 0.7	−0.9
Number of arterial grafts	1.8 ± 0.9	1.5 ± 0.7	33.8	1.8 ± 0.9	1.8 ± 0.8	4.3
Graft targets						
Arterial to anterior wall (LAD, diagonal [D], LM)	774 (98.1%)	2162 (98.0%)	0.4	98.1%	98.5%	−3.2
Arterial to lateral wall (OM, circumflex, ramus [RI])	349 (44.2%)	836 (37.9%)	12.9	44.2%	45.0%	−1.6
Arterial to inferior wall (RCA, posterior descending [PDA], posterior lateral [PL])	22 (2.8%)	57 (2.6%)	1.3	2.8%	2.6%	1.0
Additional SVG to anterior wall (LAD, diagonal [D], LM)	134 (17.0%)	563 (25.5%)	−21.0	17.0%	19.1%	−5.4
SVG to lateral wall (OM, circumflex, ramus [RI])	363 (46.0%)	1367 (62.0%)	−32.5	46.0%	48.1%	−4.2
SVG to inferior wall (RCA, posterior descending [PDA], posterior lateral [PL])	665 (84.3%)	1711 (77.6%)	17.1	84.3%	82.8%	4.0
Medical therapy at discharge						
Aspirin, n (%)	747 (94.7%)	2103 (95.4%)	−3.2	94.7%	94.0%	3.0
Beta-blocker, n (%)	653 (82.8%)	1846 (83.7%)	−2.6	82.8%	79.2%	9.2
Statin, n (%)	723 (91.6%)	2064 (93.6%)	−7.5	91.6%	88.7%	9.9
Clopidogrel, n (%)	152 (19.3%)	460 (20.9%)	−4.0	19.3%	17.8%	3.7

*VEST*, Venous external support; *ART*, Arterial Revascularization Trial; *SD*, standard deviation; *BMI*, body mass index; *CABG*, coronary artery bypass grafting; *LAD*, left anterior descending; *LM*, left main; *OM*, obtuse marginal; *R1*, ramus intermedius; *RCA*, right coronary artery.

### Major Adverse Cardiac and Cerebrovascular Events

**Figure 2:**
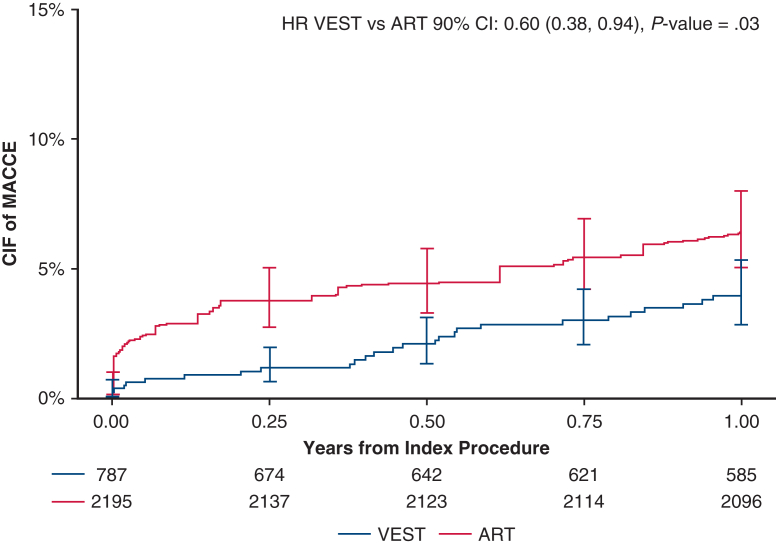
Weighted cumulative incidence curves of MACCE (major adverse cardiovascular and cerebrovascular events including all-cause mortality, myocardial infarction, repeat revascularization, and cerebrovascular accident) between treatment groups through 1 year after coronary artery bypass grafting. *VEST*, Venous external support; *ART*, Arterial Revascularization Trial; *HR*, hazard ratio; *CIF*, cumulative incidence function.

At 1-year post-CABG, the weighted cumulative incidence of MACCE was 4.13% in the VEST cohort and 6.53% in the ART cohort (HR, 0.60; 90% CI, 0.38-0.94, *P* = .03), with an absolute risk difference of −2.40% (90% CI, −4.80% to 0.01%, *P* = .05). The unweighted cumulative incidence of MACCE at 1 year was 4.13% in the VEST cohort and 4.99% in the ART cohort (HR, 0.78; 90% CI, 0.55-1.11, *P* = .12). Cumulative incidence curves for MACCE at 1-year post CABG surgery are presented in Figure 2.

**Figure 3:**
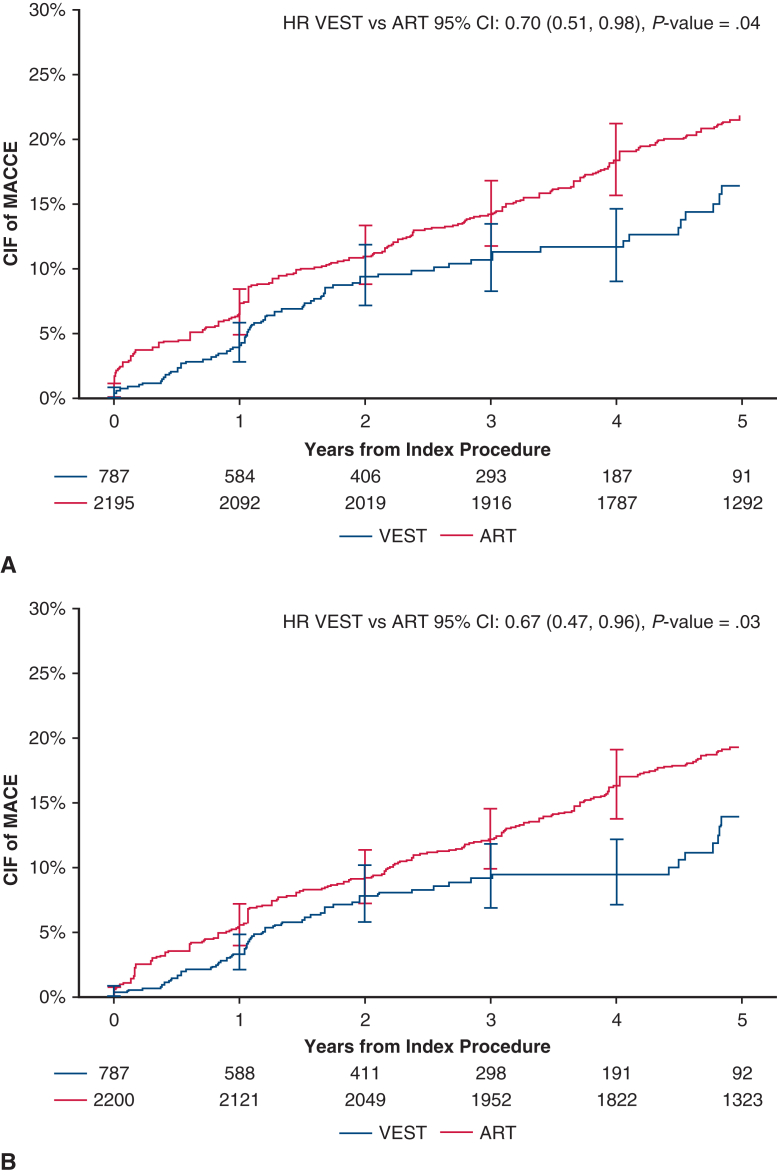
Weighted cumulative incidence of (A) MACCE (major adverse cardiovascular and cerebrovascular events including all-cause mortality, myocardial infarction, repeat revascularization, and cerebrovascular accident), and (B) MACE (major adverse cardiovascular events including all-cause mortality, myocardial infarction, repeat revascularization) between treatment groups, through 5 years. *VEST*, Venous external support; *ART*, Arterial Revascularization Trial; *HR*, hazard ratio; *CIF*, cumulative incidence function.

**Figure 4:**
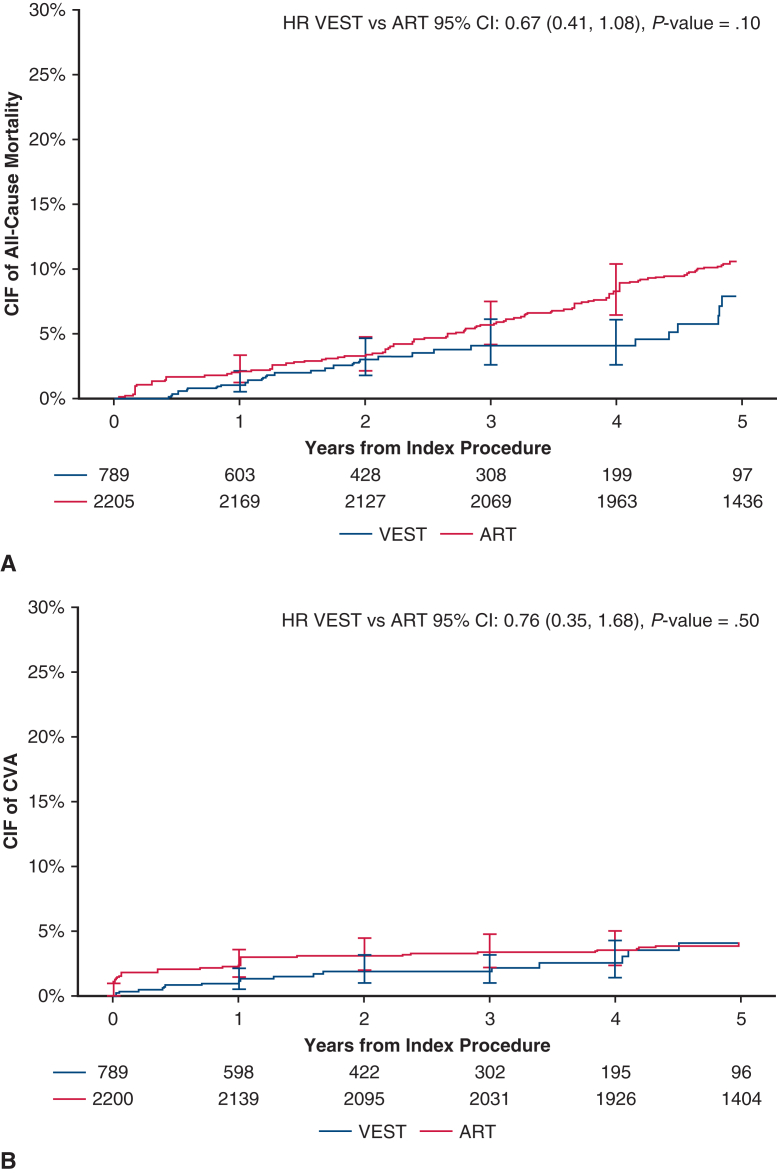

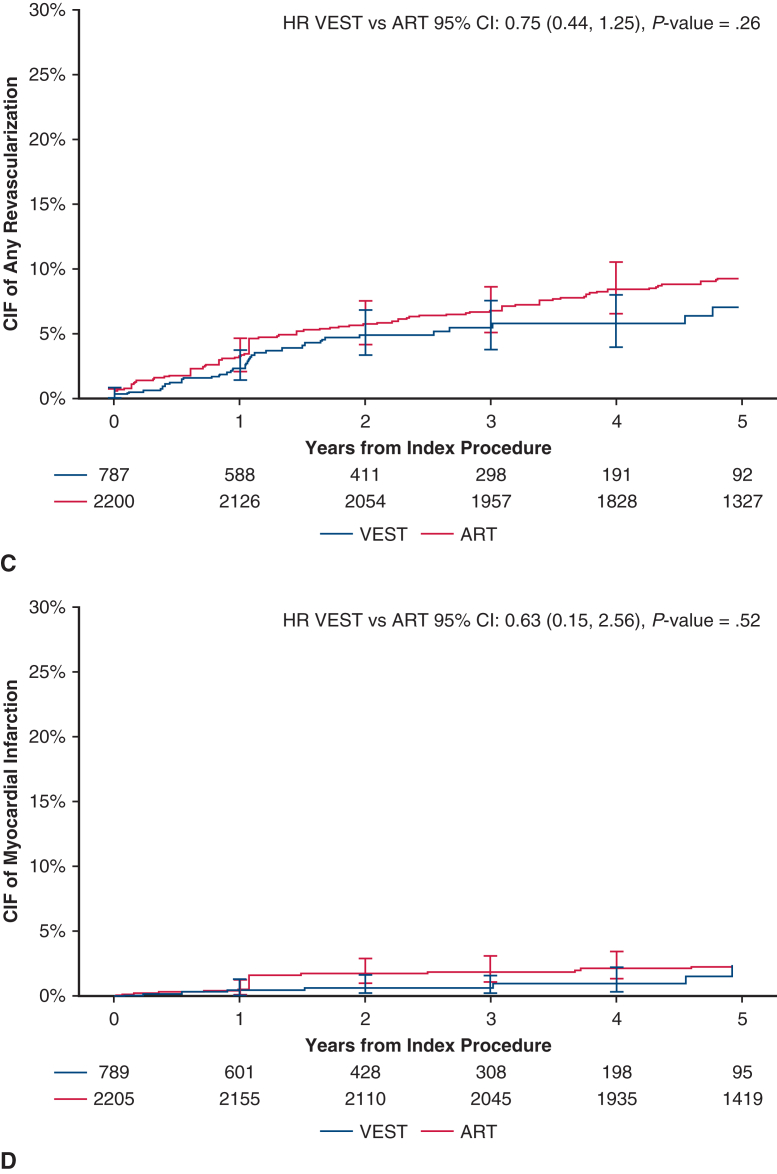
Weighted cumulative incidence curves of MACCE (major adverse cardiovascular and cerebrovascular events) components between treatment groups: (A) all-cause mortality; (B) cerebrovascular accident (*CVA*); (C) any revascularization; (D) myocardial infarction. *VEST*, Venous external support; *ART*, Arterial Revascularization Trial; *HR*, hazard ratio; *CIF*, cumulative incidence function.

**Figure 5:**
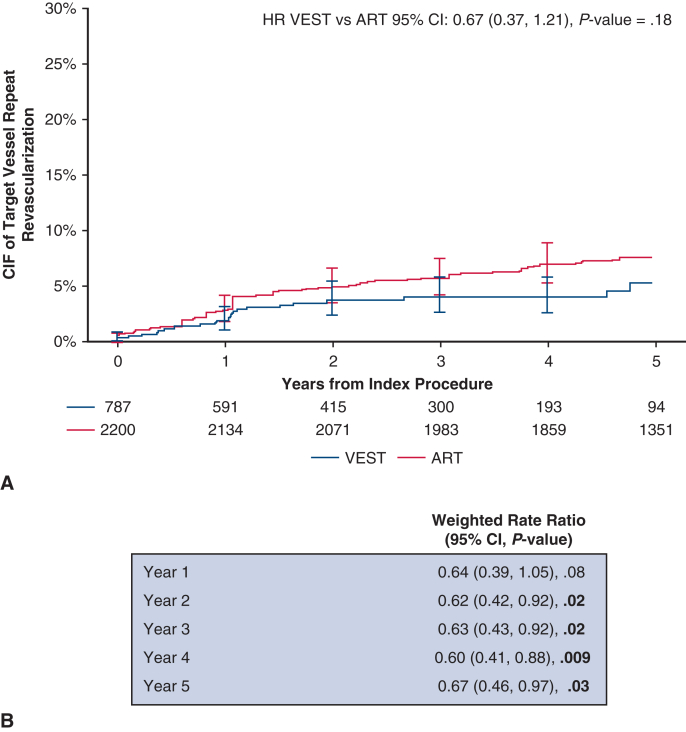
Target vessel revascularization (*TVR*): (A) weighted cumulative incidence curves to assess overall differences in time-to-first-TVR; and (B) weighted annual rate analysis using Poisson regression to compare TVR rates per person-year at each time interval. *VEST*, Venous external support; *ART*, Arterial Revascularization Trial; *HR*, hazard ratio; *CIF*, cumulative incidence function; *CI*, confidence interval.

Over 5 years post-CABG, with a median follow-up of 736 days in VEST and 1825 days (5 years) in ART, the weighted cumulative incidence of MACCE at 5 years was 16.43% in VEST and 21.82% in ART (HR, 0.70; 95% CI, 0.51-0.98; *P* = .04, Table 2), with an absolute risk difference of −5.40% (95% CI, −11.58%-0.79%; *P* = .09). The unweighted cumulative incidence of MACCE was 16.43% in VEST and 17.52% in ART (HR, 0.92; 95% CI, 0.71-1.19; *P* = .52). The weighted cumulative incidence of MACE at 5 years was 13.91% in the VEST cohort and 19.31% in the ART cohort (HR, 0.67; 95% CI, 0.47-0.96, *P* = .03, Table 2). The unweighted HR for MACE at 5 years was 0.88 (95% CI, 0.67-1.17, *P* = .38). Cumulative incidence curves for MACCE and MACE are presented in Figure 3. The cumulative incidence of individual MACCE components through 5 years is presented in Figure 4. After weighting, annual TVR rates per person-year were significantly lower in the VEST cohort compared with ART at 2, 3, 4, and 5 years with respective *P* values of .02, .02, .009, and .03 (Figure 5).

**Table 2:** Weighted cumulative incidence of clinical events at 5 years

Clinical outcome	Cumulative incidence of events Patients in VEST, % (n = 789)	Cumulative incidence of events Patients in ART, % (n = 2205)	Weighted HR (95% CI), *P* value
MACCE at 5 y	16.43	21.82	0.70 (0.51-0.98), **.04**
MACE at 5 y	13.91%	19.31%	0.67 (0.47, 0.96), **.03**
All-cause mortality at 5 y	7.88%	10.59%	0.67 (0.41-1.08), .10
Cerebrovascular accident at 5 y	4.13%	4.14%	0.76 (0.35-1.68), .50
Any revascularization at 5 y	7.07%	9.27%	0.75 (0.44-1.25), .26
Myocardial infarction at 5 y	2.34%	2.28%	0.63 (0.15-2.56), .52

Bold values indicate statistically significant.

*VEST*, Venous external support; *ART*, Arterial Revascularization Trial; *HR*, hazard ratio; *CI*, confidence interval; *MACCE*, major adverse cardiovascular and cerebrovascular events; *MACE*, major adverse cardiovascular events.

## Discussion

This propensity-matched analysis shows that use of external SVG stenting for CABG is associated with significant reduction of MACCE at 1 and 5 years after CABG, driven at least partially, by significant reduction in vein graft target revascularization. Progressive vein graft disease remains a major limitation of CABG. SVGs are inherently susceptible to structural degeneration over time. Previous studies have demonstrated that external stenting can attenuate disease progression by significantly reducing SVG intimal hyperplasia and promoting lumen uniformity.^19^^,^^20^ In addition, these pathologic changes have been associated with reductions in MACCE.^21^ However, the direct impact of external SVG stenting compared with standard surgical approaches on long-term clinical outcomes has not been previously investigated. This study represents the largest comparative analysis of external stenting and is the first adequately powered analysis to evaluate its effects on clinical outcomes.

In this study, we evaluated the impact of external stenting by comparing outcomes of real-world patients treated with VEST to those of the ART study cohort. ART,^2^ with its predominantly European patient population and relatively low incidence of MACCE compared to other contemporary large-scale CABG trials,^22^, ^23^, ^24^, ^25^, ^26^, ^27^, ^28^, ^29^ served as a rigorous comparator. A propensity score−matched analysis was used to adjust for potential confounding factors, including demographic, baseline clinical characteristics, surgical variables, and postoperative medication use.

We observed a significant reduction in MACCE at 1 year, with this benefit persisting through to 5 years. Notably, a reduction in MACCE was observed early in the follow-up period, consistent with the findings of Dushaj and colleagues^5^ who reported that external stenting reduces perioperative thrombotic vein graft occlusion, potentially as the result of its kink-resistant properties.

Although composite MACCE provides a comprehensive measure of clinical outcomes after CABG, external stenting is not expected to influence the incidence of stroke due to its mode of action and implantation site. Two key observations from this study reinforce this understanding. First, the incidence of CVAs did not differ between the treatment and control groups over 5 years. Consequently, the HR for MACE over this period appeared further improved relative to composite MACCE. In addition, the benefits of external stenting on revascularization and myocardial infarction increased progressively over time.

To more precisely assess the direct impact of external stenting, we analyzed TVR. Although the broader revascularization end point includes interventions performed on both previously grafted and de novo coronary territories, TVR specifically accounts for interventions targeting previously vein grafted regions, which may be directly influenced by external stenting. Our findings demonstrated that TVR rates per person-year were significantly lower in the VEST cohort at 2 to 5 years and are consistent with previous randomized trials that demonstrated reduction in ischemic driven revascularization in externally stented vein grafts.^20^^,^^30^

Despite differing time periods, it is reassuring that postoperative medication, which is very critical to long term outcomes, was very similar in both groups. Although not randomized comparisons, this is consistent with propensity matching suggesting that there were no systematic differences in postoperative treatment between the 2 groups.

### Limitations

Our analysis has some limitations. As a comparative observational analysis, it is subject to potential biases, both known and unknown, despite the use of rigorous propensity score matching to balance baseline covariates and patient management variables. In particular, there is reassurance in the almost identical postoperative pharmacological management of patients.

In addition, the ART trial was a randomized controlled study that enrolled patients nearly 2 decades ago, whereas the VEST cohort comprises real-world patients treated in a more contemporary clinical setting, which may introduce differences in practice patterns and treatment approaches. Although surgical techniques and pharmacologic management associated with CABG have remained largely consistent over this period, advancements in perioperative and postoperative care along with updated practice guidelines, may influence patient outcomes. Propensity score adjustment cannot typically account for time-dependent confounding or practice evolution. The inclusion of surgical practice and disease management variables, such as pump use or concomitant procedures, mitigated some of this uncertainty. Detailed vein harvesting and storage data is not available in the ART database, however harvesting techniques are unlikely to affect clinical outcomes.^31^ Further, as reflected in the baseline demographic characteristics of our study, these developments may be counterbalanced by the increasing complexity of contemporary patients who undergo CABG, who present with a greater burden of comorbidities, compared with those in the ART trial. This trend is likely attributable to the growing role of PCIs, which have increasingly been used in patients with less-complex coronary artery disease. In addition, it is widely recognized that randomized trials typically enroll lower-risk patients compared with real-world populations, suggesting that outcomes in the ART cohort would be more favorable than those in the real-world VEST cohort. However, this was not observed in our analysis, further supporting the potential clinical benefits of VEST. Lastly, low event rates for secondary end points such as MI and CVA resulted in wide confidence intervals and limited power to detect meaningful differences.

## Conclusions

External SVG stenting has the potential to improve clinical outcomes to 5 years after CABG. This effect appears to be closely linked to the previously established benefits of external stenting, including attenuation of SVG disease progression and improved kink resistance. External SVG stenting can now be considered as a potentially effective strategy to significantly improve patient outcomes after CABG.

### Webcast

You can watch a Webcast of this AATS meeting presentation by going to: https://www.aats.org/resources/comparative-study-of-long-term-10278.

**Figure:**
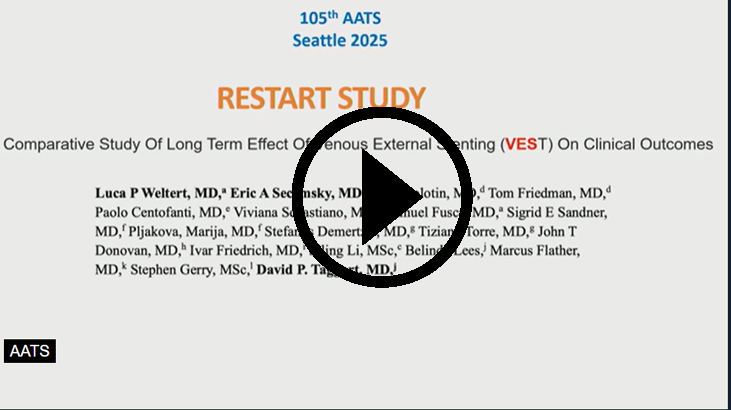

### Audio

You can listen to the discussion audio of this article by going to the supplementary material section below.

## Conflict of Interest Statement

Profs Taggart and Bolotin hold share options with Vascular Graft Solutions. All other authors reported no conflicts of interest.

The *Journal* policy requires editors and reviewers to disclose conflicts of interest and to decline handling or reviewing manuscripts for which they may have a conflict of interest. The editors and reviewers of this article have no conflicts of interest.

## Supplementary Data

### Audio

Audio 1 This is an audio recording of the presentation of the associated abstract and discussion. The abstract presentation ends at 12:32 and the discussion begins at 12:36.

Video 1 Video available at: https://www.jtcvs.org/article/S2666-2736(25)00379-1/fulltext.
